# Genetic polymorphisms associated with increased risk of developing chronic myelogenous leukemia

**DOI:** 10.18632/oncotarget.5915

**Published:** 2015-10-12

**Authors:** Heriberto Bruzzoni-Giovanelli, Juan R. González, François Sigaux, Bruno O. Villoutreix, Jean Michel Cayuela, Joëlle Guilhot, Claude Preudhomme, François Guilhot, Jean-Luc Poyet, Philippe Rousselot

**Affiliations:** ^1^ Université Paris Diderot, Sorbonne Paris Cité UMRS 1160 INSERM, Paris, France; ^2^ Centre d'Investigations Cliniques 9504 INSERM-AP-HP Hôpital Saint-Louis, Paris, France; ^3^ Centre de Recerca en Epidemiologia Ambiental (CREAL), Barcelona, Spain; ^4^ Institut Municipal d'Investigació Mèdica (IMIM), Barcelona, Spain; ^5^ CIBER Epidemiología y Salud Pública (CIBERESP), Spain Centre de Recerca en Epidemiologia Ambiental (CREAL), Barcelona, Spain; ^6^ Institut Universitaire d'Hématologie, Université Paris Diderot, Sorbonne Paris Cité, Paris, France; ^7^ Université Paris Diderot, Sorbonne Paris Cité UMRS 973 Inserm, Paris, France/Inserm, U973, Paris, France; ^8^ Laboratoire Central d'Hématologie, Hôpital Saint Louis, Paris, France; ^9^ EA3518, Université Paris Diderot, Sorbonne Paris Cité, Paris, France; ^10^ Inserm CIC 0802, CHU de Poitiers, Poitiers, France; ^11^ Laboratoire d'Hématologie, Inserm, U837, CHRU, Lille, France/Université de Lille Nord, Institut de Recherche sur le Cancer de Lille, Lille, France; ^12^ Service d'Hématologie et d'Oncologie, Hôpital Mignot, Université Versailles, Saint-Quentin-en-Yvelines, France

**Keywords:** CML, SNPs, genetic predisposition, myeloid leukemia

## Abstract

Little is known about inherited factors associated with the risk of developing chronic myelogenous leukemia (CML). We used a dedicated DNA chip containing 16 561 single nucleotide polymorphisms (SNPs) covering 1 916 candidate genes to analyze 437 CML patients and 1 144 healthy control individuals. Single SNP association analysis identified 139 SNPs that passed multiple comparisons (1% false discovery rate). The HDAC9, AVEN, SEMA3C, IKBKB, GSTA3, RIPK1 and FGF2 genes were each represented by three SNPs, the PSM family by four SNPs and the SLC15A1 gene by six. Haplotype analysis showed that certain combinations of rare alleles of these genes increased the risk of developing CML by more than two or three-fold. A classification tree model identified five SNPs belonging to the genes PSMB10, TNFRSF10D, PSMB2, PPARD and CYP26B1, which were associated with CML predisposition. A CML-risk-allele score was created using these five SNPs. This score was accurate for discriminating CML status (AUC: 0.61, 95%CI: 0.58–0.64). Interestingly, the score was associated with age at diagnosis and the average number of risk alleles was significantly higher in younger patients. The risk-allele score showed the same distribution in the general population (HapMap CEU samples) as in our control individuals and was associated with differential gene expression patterns of two genes (VAPA and TDRKH). In conclusion, we describe haplotypes and a genetic score that are significantly associated with a predisposition to develop CML. The SNPs identified will also serve to drive fundamental research on the putative role of these genes in CML development.

## INTRODUCTION

Although chronic myelogenous leukemia (CML) is the first example of a leukemia linked to a single cytogenetic and molecular lesion (the chimeric BCR-ABL protein with constitutive tyrosine kinase activity resulting from the t(9;22) translocation), little is known regarding putative inherited factors associated with the risk of developing this disease. Familial cases of CML are infrequent [1] suggesting that the contribution of multiple genes may be necessary to define a pattern of genetic predisposition.

Association studies with gene candidates have suggested that single nucleotide polymorphisms (SNPs) may be related to CML progression [2, 3] and to response to therapy [4-11]. There is some evidence in the literature, albeit patchy, reporting an association between various SNPs and the risk of developing CML. Kim et al found that BCL2 SNPs were associated with an increased risk of developing CML [12]. In a Chinese population, the G/G genotype in SNP309 of MDM2 [13] and two SNPs in the 5′ regulatory region of the PDCD5 gene [14] appear to impact CML predisposition. A possible association of CML susceptibility with SNPs of the CYP1A1 and GSTT1 genes [15] or the GSTM1 and GSTT1 genes [16] has also been reported, although these polymorphisms appear to be restricted to Turkish and Indian populations. Nonetheless, another Chinese study failed to find any association with CML predisposition among SNPs in CYP1A1, CYP2D6, GSTM1 and GSTT1 genes [17]. A possible association between polymorphisms in KIR2DS4 [18] or MTHFR genes [19] and CML predisposition has been suggested. Finally, a genome-wide association study identified two loci (6q25.1 and 17p11.1) associated with susceptibility to CML [20].

The complete lack of data in this field concerning the Caucasian population is striking. Here we present an SNP association study conducted using DNA samples from 437 French CML patients compared to 1 144 healthy French control individuals. CML-risk haplotypes were identified and a genetic risk score was developed and validated using re-sampling techniques.

## RESULTS

### Population characteristics

Median age and sex ratio distribution were compared between the two groups (Table 1). Sex distribution was comparable between patients and controls, as were distribution of age and sex within each group. The median age of individuals in the control group was 10 years older than in the CML cohort, and analyses were thus adjusted for age. The distribution of the Sokal score in CML patients was 40.4 % low, 39.8 % intermediate, and 19.9 % high.

**Table 1:** Population characteristics

	Control N=1 144)		CML (N=437)	
	N (%)	Median age (years)	N (%)	Median age (years)
N men	675 (59%)	64.7	259 (60%)	55.1
N women	469 (41%)	64.9	174 (40%)	55.4
p-value		0.795		0.862

### Single SNP association analyses

Overall, 13 937 out of 16 561 SNPs (84.1 %) were well genotyped in all evaluated participants (CML patients and controls) and passed quality control criteria. Principal component analysis was performed and no stratification was observed (Supplemental data Figure S1).

**Figure 1:**
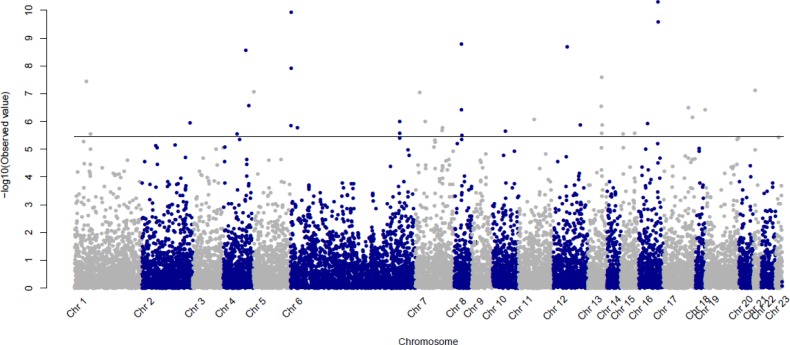
Manhattan Plot presenting p-values (−log10 scale) for the association between CML patients and controls for the 13 937 SNPs passing quality control criteria The horizontal line indicates the 1% FDR significance threshold.

Single association analysis identified 139 SNPs that were significantly associated with CML at an FDR < 0.01 (Figure 1 and Supplemental data in Table S1). We observed that among the 139 SNPs, 11 correspond to genes coding for detoxification proteins and 12 to genes coding for transporter proteins (two families well represented in the DNA chip) [6].

Among the genes listed in Table S1, eight are represented by three or more SNPs (Supplemental data Table S2). Three SNPs are associated with the histone deacetylase HDAC9, the caspase activation inhibitor AVEN, the semaphorin SEMA3C, the inhibitor of kappaB kinase beta (IKBKB), the glutathione S-transferase GSTA3, the receptor TNFRSF-interacting serine-threonine kinase 1 (RIPK1) and the growth factor FGF2. Four SNPs belong to the immunoproteasome subunits (PSMB2, PSMA8 and PSMB10) and six SNPs are located in the SLC15A1 gene, a solute carrier of di- and tripeptides.

Haplotype analyses were performed for the eight genes from Table S1 which are represented by three or more SNPs (Supplemental data Table S3). Several haplotypes containing rare alleles for these SNPs were associated with increased CML risk compared to the reference haplotypes. These reference haplotypes (determined by the most frequent alleles of each SNP) are present at frequencies ranging from 47.39 % to 89.44 % in the general population. Haplotype analysis of three SNPs located in the SLC15A1 gene (rs6491437, rs950905, rs9557033) showed an increased risk for people having haplotypes containing two (BAB) or three rare alleles (BBB) with respect to the reference haplotype (OR = 2.23 and 2.00, respectively, *p* < 10^−3^). An increased risk of CML was also observed for individuals with haplotypes containing three rare alleles for the AVEN gene (rs7182969, rs527834, rs2632075; OR = 3.16, *p* < 10^−3^), the SEMA3C gene (rs6978637, rs10261267, rs17147989; OR = 2.75, *p* < 10^−3^), the IKBKB gene (rs11986055, rs4560769, rs6474386; OR = 2.61, *p* < 10^−3^), the GSTA3 gene (rs512795, rs2281594, rs9296695; OR = 2.12, *p* < 10^−3^), the RIPK1 gene (rs2077681, rs9392454, rs4959774, OR = 2.15, *p* < 10^−3^), and the FGF2 gene (rs3804158, rs10452197, rs308388, OR = 1.62, *p* < 10^−3^). For the HDAC9 gene (rs3852253, rs801540, rs6958865), increased risk was observed in the presence of the rare allele for rs3852253 and the common allele for the SNP rs801540 (OR = 2.23 and 3.03, respectively, *p* < 10^−3^) (Supplemental data Table S3). Moreover, haplotypes of PSMA8 with two SNPs (rs4800723 and rs895630) were analyzed and rare alleles were also associated with an increased CML risk (OR = 1.83, *p* < 10^−3^).

### Genetic score

**Figure 2:**
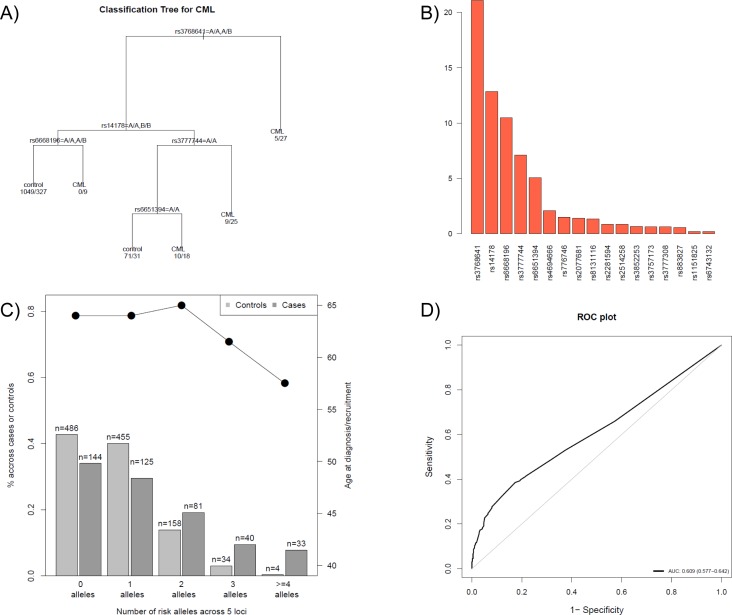
SNP selection procedure and prediction of the probability of developing CML **A**)A classification tree was developed to discriminate CML versus control individuals, using the SNPs obtained from a stepwise multivariate logistic model from the 139 selected SNPs in the single analysis. As an example, individuals carrying two copies of a minor allele B of SNP rs376864 are more likely to develop CML than individuals carrying the normal allele and hence were classified as CML patients. For individuals carrying at least one normal allele, the SNP rs14178 was also taken into account; individuals having normal alleles for rs14178 and rs6668196 were classified as controls, however, individuals having a normal allele for rs14178 and two rare alleles for rs6668196 were classified as CML. **B**) Variable importance plot used to create the classification tree. The barplot shows a large decay of importance from the sixth SNP, so only the first five SNPs were used to classify individuals (i.e. in the classification tree in A). **C**) Distribution of the genetic score (sum of risk alleles) created using the five selected SNPs obtained from the classification tree methodology. **D**) ROC curve and its AUC value.

Using a classification tree approach (Figure 2A) and its variable importance plot (Figure 2B), SNPs were selected for predicting the probability of developing CML. Table 2 shows the five SNPs which were identified using a multivariate logistic model following the classification tree approach [27] including the 139 SNPs that were associated with CML in the single analysis. The five SNPs identified were rs14178, rs6651394, rs6668196, rs3777744 and rs3768641 and belong to genes PSMB10, TNFRSF10D, PSMB2, PPARD and CYP26B1, respectively. The regional plots of these selected SNPs are shown in Supplemental data Figure S2.

**Table 2:** SNPs associated with increased likelihood of developing CML, identified by multivariate logistic regression following the classification tree approach The 139 SNPs identified in the single SNP association analysis were used.

SNP risk alleles	Odds Ratio	95% CI	p-value	Associated Gene
rs14178	2.31	(1.71 – 3.11)	4.65 × 10-8	PSMB10
rs6651394	1.65	(1.30 – 2.08)	3.31 × 10-5	TNFRSF10D
rs6668196	1.61	(1.20 – 2.15)	1.35 × 10-3	PSMB2
rs3777744	1.43	(1.13 – 1.81)	2.70 × 10-3	PPARD
rs3768641	1.42	(1.11 – 1.82)	5.04 × 10-3	CYP26B1

**Figure 3:**
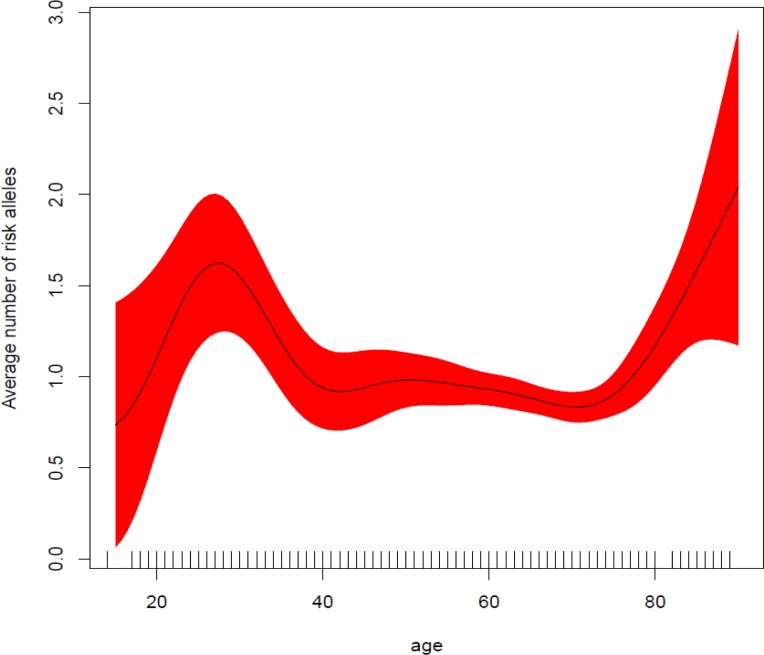
Average number of risk alleles as a function of age at diagnosis Genetic risk alleles are more frequent in younger patients, with risk starting to increase again for older patients (>75 years). The X-axis depicts the number of risk alleles grouped in different categories (0, 1, 2, 3, +4 alleles). The right Y-axis represents the number of individuals (bars) while the left Y-axis shows the mean age (dots) for each risk score category.

The distribution of the genetic score created using these five SNPs in patients and controls as well as its association with age at diagnosis are depicted in Figure 2C. We observed that the risk of developing CML increased with the number of risk alleles (per-allele OR=1.61, 95% CI 1.45 to 1.80, *p* = 1.7×10^−18^). Younger patients diagnosed with CML, had a higher number of risk alleles (Figure 3). Interestingly, the importance of these risk alleles was also increased for the oldest CML patients. The risk score showed a discriminating power of 61% (AUC = 0.609, 95% CI 0.577 to 0.642) (Figure 2D). The multivariate model including the five SNPs contributing to the genetic score accounts for 8.2 % of the total variability in CML patients.

### Transcriptomic analysis

No differences between genetic risk score were observed among the 105 CEU individuals and our controls (p = 0.4456, data not shown). To characterize the possible functional consequences of the CML genetic risk score, we analyzed gene expression levels in lymphoblastoid cell lines of HapMap CEU samples. Several genes located on different chromosomes were identified among the top ten differentially expressed genes per risk allele increase (Supplemental data Table S4). In particular, VAPA (vesicle-associated membrane protein-associated protein A) and TDRKH (Tudor and KH domain-containing protein) expression was highly correlated with the number of risk alleles (*p* = ~10^−7^). Enrichment analysis based on the GO and KEGG databases was performed including all genes differentially expressed with respect to the CML genetic score (*p* < 10^−3^) and several GO and KEGG terms were associated with genetic risk score (Supplemental data Table S4).

## DISCUSSION

The clinical significance of the studies performed to date, notably in a European context, suggesting a link between predisposition to CML and SNPs is far from being established; firstly because most of these studies have been performed using very few candidate genes, secondly because the gene sets analyzed were different across studies, and thirdly because the same SNPs were not analyzed for the same gene. Kim et al have performed the only published study analyzing a large number of SNPs in relation to CML susceptibility, reporting the association of two loci, although only 17p11.1 was validated, and then only in a Korean population [20].

We report here a case-control study investigating genetic polymorphisms associated with the risk of developing CML. Overall, 437 CML patients and 1 144 controls were genotyped using a custom-made DNA chip containing 16 561 SNPs. We identified 139 SNPs which were significantly associated with the risk of developing CML (FDR < 0.01). Although patient age and Sokal score distribution of our CML cohort were representative of CML disease epidemiology, no significant SNP associations were identified in relation to the Sokal score, suggesting it is more closely related to tumor characteristics than to the constitutional DNA.

For nine genes represented by at least three SNPs each from the 139 SNPs, we identified haplotypes composed of rare alleles which are associated with an increased risk of developing CML (up to three-fold relative to the reference haplotypes). For instance, for the SLC15A1 gene the presence of two rare alleles with a total frequency of 14.39 % (4.72 % + 9.67 %), doubled the risk of developing CML compared to the reference haplotype (which is present in half of the general population, 47.39 %). Similar results were found for haplotypes containing three rare alleles for the AVEN, SEMA3C, IKBKB, GSTA3, RIPK1 and the FGF2 genes, with frequencies ranging from 2.25% to 12.9 %. In the case of HDAC9, an increased risk was observed for haplotypes associating the rare allele for the SNP rs3852253 and the common allele for rs801540.

A classification tree approach was used to in order to select SNPs that better predict the probability of developing CML. This approach allowed us to identify five SNPs belonging to the PSMB10, TNFRSF10D, PSMB2, PPARD and CYP26B1 genes, which we used to create a genetic CML-risk-allele score with a high discriminating power (61%; 95% CI 58% to 64%) and a multivariate model accounting for 8.2% of the total variability observed in CML patients. Ideally, an independent sample should be used for validating purposes. As the DNA chip was not commercially available, we were only able to analyze patients while this chip was available. We thus decided to pool all of the patients in a single cohort. In order to reduce false discoveries and to address this limitation, our method was based on selecting SNPs that pass a more stringent FDR threshold than is typically used in such studies (i.e., 1% *vs* 5% as is usually set in genome-wide association studies).

The discriminating power of our proposed score was supported by two pieces of evidence: its distribution as a function of age at CML diagnosis and its representation in the reference CEU population. The importance of the genetic score differed with age at diagnosis; we found an inverse relationship between the number of risk-alleles and age. On the basis of these results, it can be speculated that the occurrence of CML in younger people may be driven by specific inherited parameters given that the average number of risk alleles is significantly higher in this sub-population. Interestingly, the importance of the number of risk alleles present appears to increase again in older patients (from 75 years). Taken together, our results suggest that for intermediate ages, the influence of environmental factors at diagnosis take on greater importance than inherited factors for CML risk. This hypothesis should be evaluated in a study of a teenage CML population. Also it is important to note that the proposed genetic score showed the same distribution in CEU samples as in our control individuals (*p* = 0.4456).

One characteristic specific to this study was the use of mainly tag-SNPs on the DNA chip. As a result, most of the identified SNPs are non-coding (only one of the six SNPs identified in SLC15A1 implicates changes in the protein sequence). Nevertheless, we cannot rule out the possibility that that the genes identified by these SNPs play a role in CML development, nor that these nucleotide changes affect gene expression (e.g. via non-coding RNA regulation). In accordance with this, a recent genome-wide association study identified four non-coding SNPs associated with myeloproliferative neoplasms other than CML (polycythemia vera, essential thrombocythemia, and primary myelofibrosis) [33]. The authors found that one of the SNPs identified is associated with reduced MYB expression in normal myeloid cells. Consistent with this, our CML-allele-risk-score also had a transcriptomic effect, strongly altering gene expression patterns of two genes (VAPA and TDRKH) in the general population (HapMap CEU samples). This suggests that combinations of SNPs belonging to the five candidate genes may lead to gene expression changes that could be related to increased CML disease predisposition.

Further studies are needed to explore the possible biological significance of the genes implicated in haplotypes, genetic score or issued from the transcriptomic analysis. In this context, it is interesting to note that in bone marrow, CYP26B1 functions as a critical regulator of all-trans retinoic acid levels essential for primitive hematopoietic cells to undergo differentiation [34], as well as of the regulatory role of TNFRSF10D in TRAIL-induced cell apoptosis [35], and that polymorphisms of PPARD are related to survival in lung cancer patients [36]. A particular focus on the PSM family of proteasome subunits identified with our approaches, may be valuable; screening for genetic abnormalities identified PSMB2 has been reported in ovarian carcinogenesis [37], while PSM gene polymorphisms are risk factors for other hematologic diseases [38]. Moreover, recognition and elimination of malignant cells by cytotoxic T lymphocytes depends on antigenic peptides generated by proteasomes. It has been established that impairment of the immunoproteasome subunits PSMB8 and PSMB10, is critical for malignant cells to escape immune recognition [39].

In conclusion this study highlights the pertinence of highly significant risk alleles identified in 437 CML patients out of 16 561 SNPs, and also provides a rationale for evaluating implicated genes for their potential role in CML development.

## PATIENTS AND METHODS

### Study population

This study was approved by the Human Ethics Committee of the Hôpital Saint-Louis, Paris, and validated by the CCTIRS and CNIL [two French information technology authorities; authorized medical research (AMR) N° 913022]. Written informed consent was obtained from all individuals prior to study participation. A total of 437 CML patients aged 18 years or older were analyzed, 189 treated at the Hôpital Saint-Louis or the Hôpital Mignot, Versailles and 248 cases from the French phase III SPIRIT clinical trial (clinicaltrials.gov: NCT00219739) [21]. The control group included 1144 unrelated and unaffected individuals aged 18 years or older from two French clinical trials in Parkinson's disease, TERRE (AMR N° 998137) and PARTAGE (AMR N° 907012) [22].

### Blood sampling and analysis

Peripheral blood samples for DNA extraction from patients and controls were collected at the time of recruitment in the respective trials. For individual genotyping we used a dedicated DNA chip (designed in 2006 by the French REPAC network) with 16 561 SNPs (mainly tag-SNPs) covering 1 916 genes (implicated in proliferation, apoptosis, immunity and metabolism [6, 22]. Genotyping was performed by Integragen SA using the Illumina GoldenGate assay. All 16 561 SNPs genotyped in the 437 CML patients and 1 144 controls were included in the quality control process. SNPs with minor allele frequency (<5%), a call rate <95% and a p-value <0.0001 with the Hardy-Weinberg equilibrium test in controls were excluded from the analysis.

### Association analysis

Departure from the Hardy-Weinberg equilibrium for all biallelic SNP markers was assessed using a fast exact test [23]. Principal component analysis was done to determine if the populations were comparable, including for ethnicity [24]. Single SNP association with CML status was performed using the likelihood ratio test based on logistic regression models using an additive mode of inheritance. The Benjamini-Hochberg method was used to control the false discovery rate (FDR) due to multiple comparisons [25]. Haplotype analysis was performed using the Haplo.stats R library [26], with a sliding windows approach to detect the optimal combination of SNPs in a selected region.

We selected SNPs that passed multiple comparisons at a 1 % FDR threshold to build the optimal model for predicting the probability of developing CML using a classification tree [27]. This method is used to predict membership of individuals (e.g. case or control) depending on SNP genotypes. It selects a set of SNPs that better predict CML status by adding new SNPs to those determined in the top branches. The hierarchical nature of its representation facilitates interpretation of how SNPs interact. We then created a CML-risk-allele score by summing the number of risk alleles (0, 1, 2) for the selected SNPs. To test the relevance of our score, we estimated the impact of having each additional risk allele by computing the odds ratio (OR) and the associated 95% confidence interval (95% CI). The distribution of the risk score was also tested in relation to age at CML diagnosis. Genetic variation in CML was estimated using the Nagelkerke R^2^ index (a pseudo-R^2^ similar to R^2^ for quantitative traits) [28]. In addition, the predictive value for CML risk was calculated as the area under the ROC curve (AUC). SNP association was assessed as a function of the Sokal score in the CML patients. Association analyses were performed using the SNPassoc R package [29], and identification of causal SNPs and pathways with ICSNPathway software.

### Gene expression analysis and gene ontology enrichment analyses

To validate a possible functional impact of the CML risk-allele score, we assessed its association with gene expression data in the general population. We used data from 105 unrelated individuals from the CEU population (Utah residents with Northern and Western European ancestry from the CEPH [“Centre d'Etudes du Polymorphisme Humain”] database) included in the HapMap, for whom RNA from lymphoblastoid cell lines is available [30]. Genomic data were obtained from the 1000 Genomes Project, and transcriptome data were downloaded from the E-MTAB-198 project from the European Bioinformatics Institute at EMBL [31]. Gene ontology (GO) and Kyoto Encyclopedia of Genes and Genomes (KEGG) databases were used for enrichment analysis, including all genes associated with the score at an FDR <10^−3^. Analyses were performed using a GOstats Bioconductor package. GO and KEGG terms were claimed as a significantly associated with the score for an FDR <0.05. Data were analyzed using R Project for Statistical Computing software (R version 3.1.2) [32].

## SUPPLEMENTARY FIGURES AND TABLES


