# Supplementary material for: Genetic polymorphisms associated with increased risk of developing chronic myelogenous leukemia
Source: Oncotarget. 2015 Oct 12;6(34):36269–77. doi: 10.18632/oncotarget.5915 (PMC4742176; doi:10.18632/oncotarget.5915)
Supplement: Supplementary file 1 [file oncotarget-06-36269-s001.pdf]

Genetic polymorphisms associated with increased risk of developing chronic myelogenous leukemia

Supplementary Material

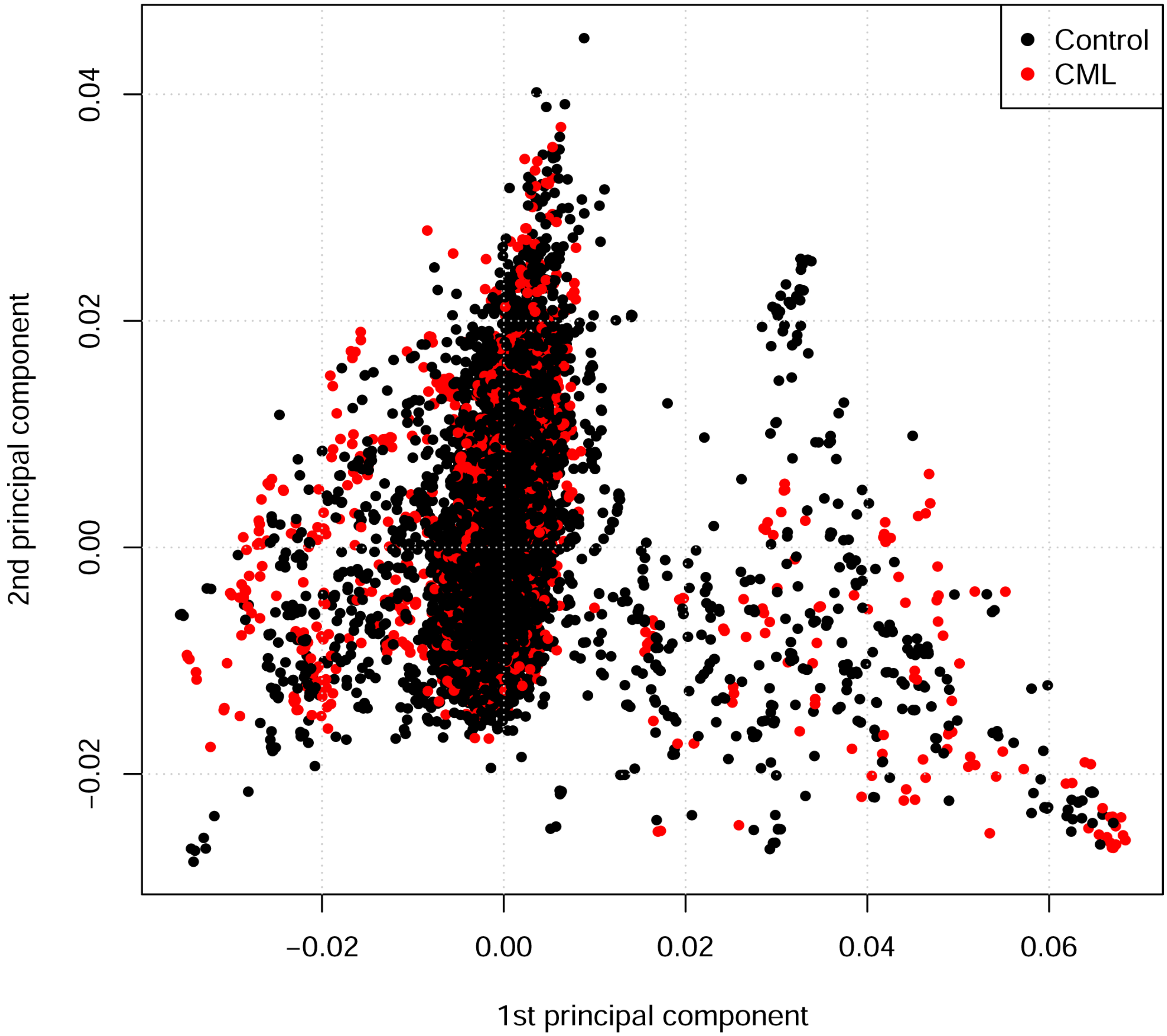

Figure S1: Principal Component Analysis plot

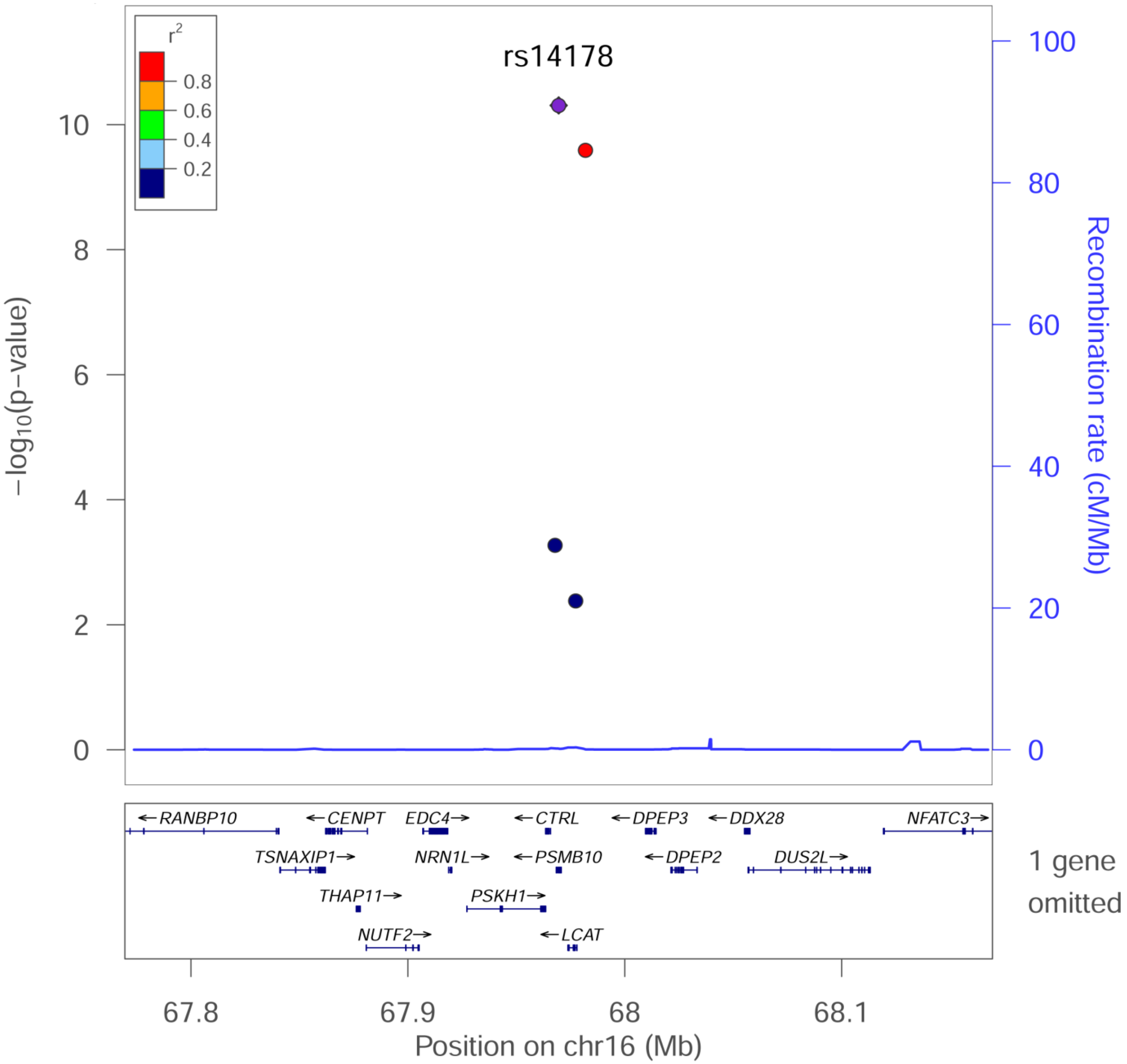
rs14178
r2
0.8
0.6
0.4
0.2
−log10(p−value)
Recombination rate (cM/Mb)
RANBP10
CENPT
EDC4
CTRL
DPEP3
DDX28
NFATC3
TSNAXIP1
NRN1L
PSMB10
DPEP2
DUS2L
THAP11
PSKH1
NUTF2
LCAT
1 gene omitted
67.8
67.9
68
68.1
Position on chr16 (Mb)

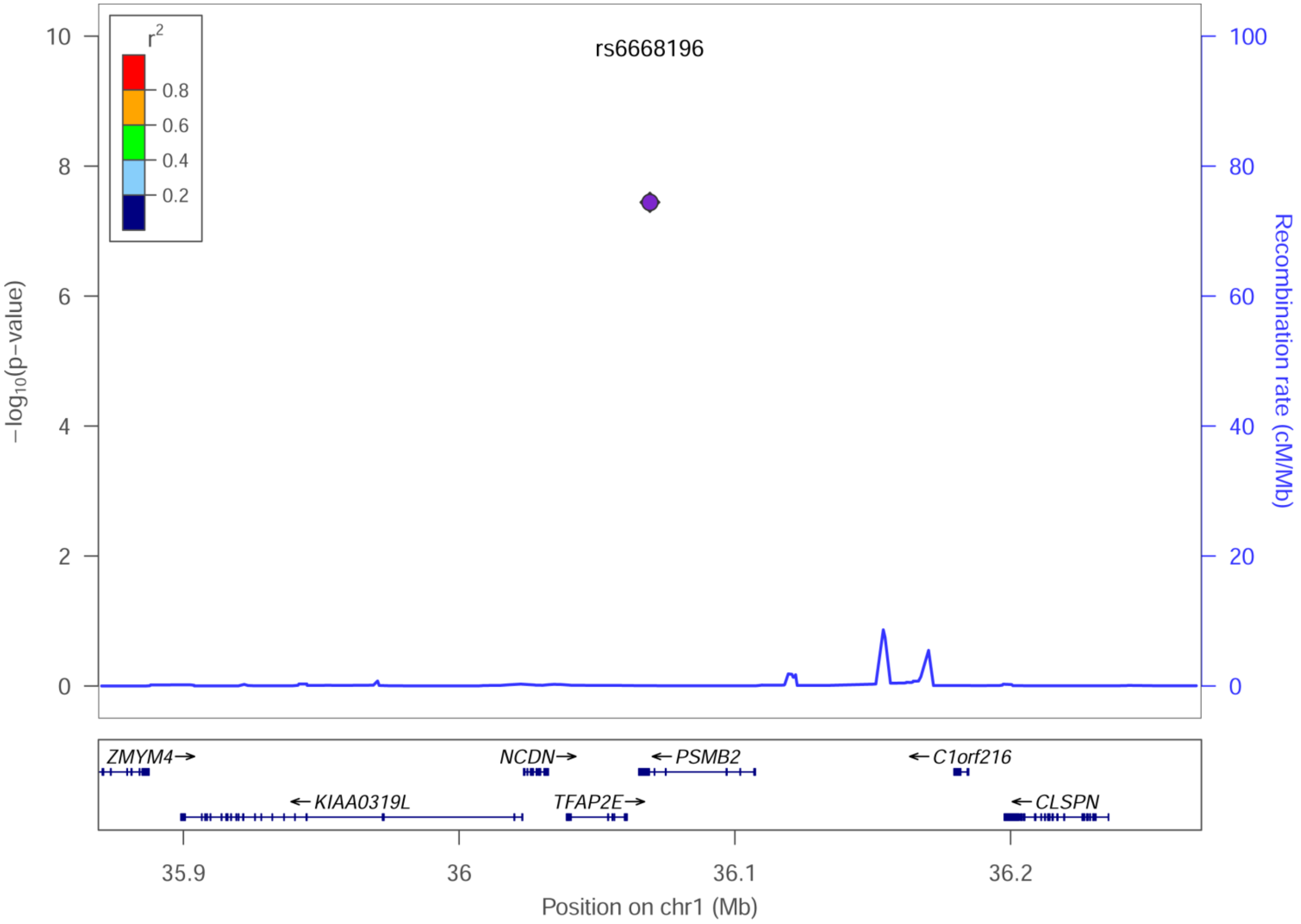

rs6668196
r2
0.8
0.6
0.4
0.2
−log10(p−value)
Recombination rate (cM/Mb)
0
2
4
6
8
10
0
20
40
60
80
100
ZMYM4→
NCDN→
←PSMB2
←C1orf216
←KIAA0319L
TFAP2E→
←CLSPN
35.9
36
36.1
36.2
Position on chr1 (Mb)

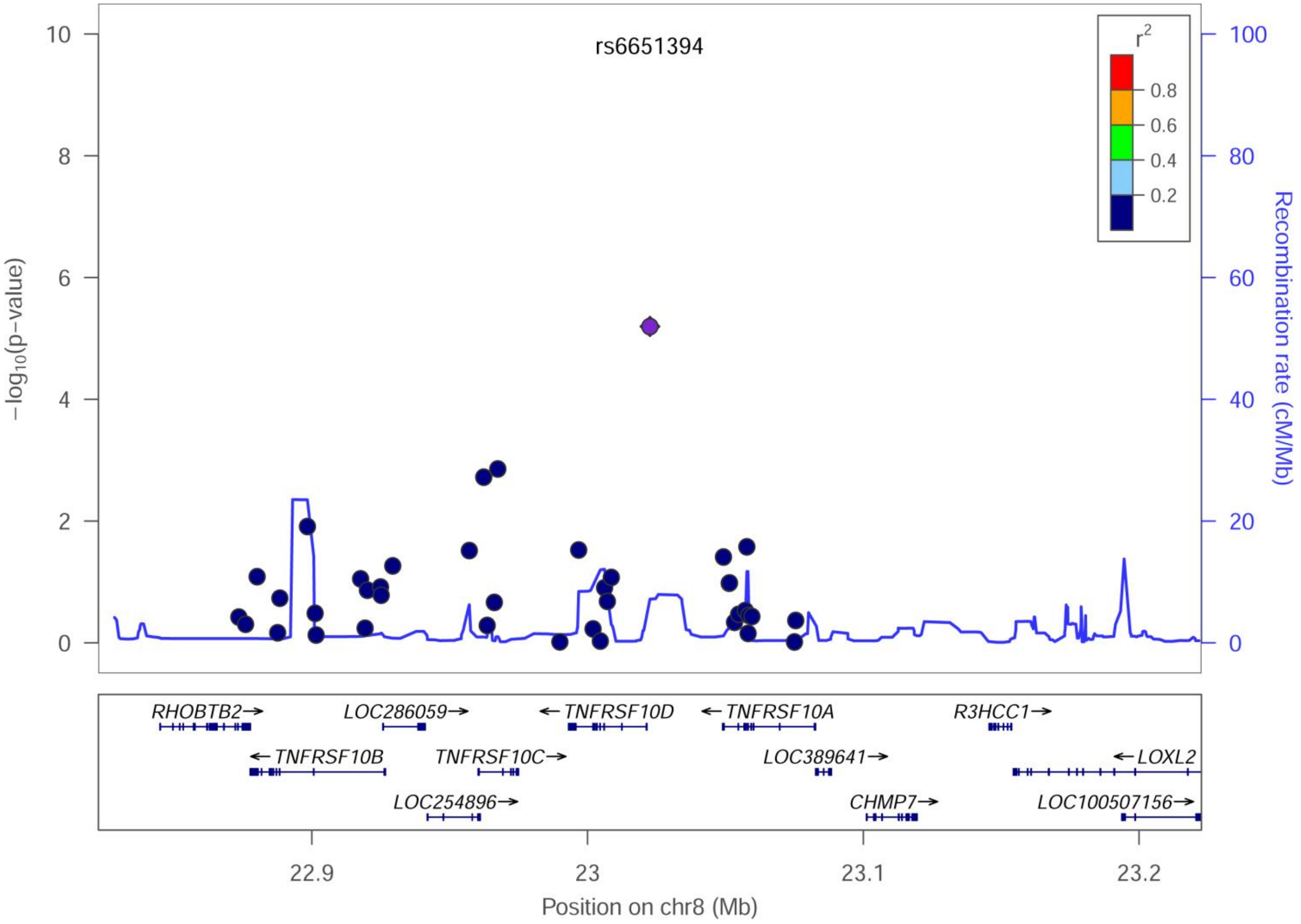

rs6651394
r2
0.8
0.6
0.4
0.2
−log10(p−value)
Recombination rate (cM/Mb)
RHOBTB2→
LOC286059→
← TNFRSF10D
← TNFRSF10A
R3HCC1→
← TNFRSF10B
TNFRSF10C→
LOC389641→
← LOXL2
LOC254896→
CHMP7→
LOC100507156→
22.9
23
23.1
23.2
Position on chr8 (Mb)

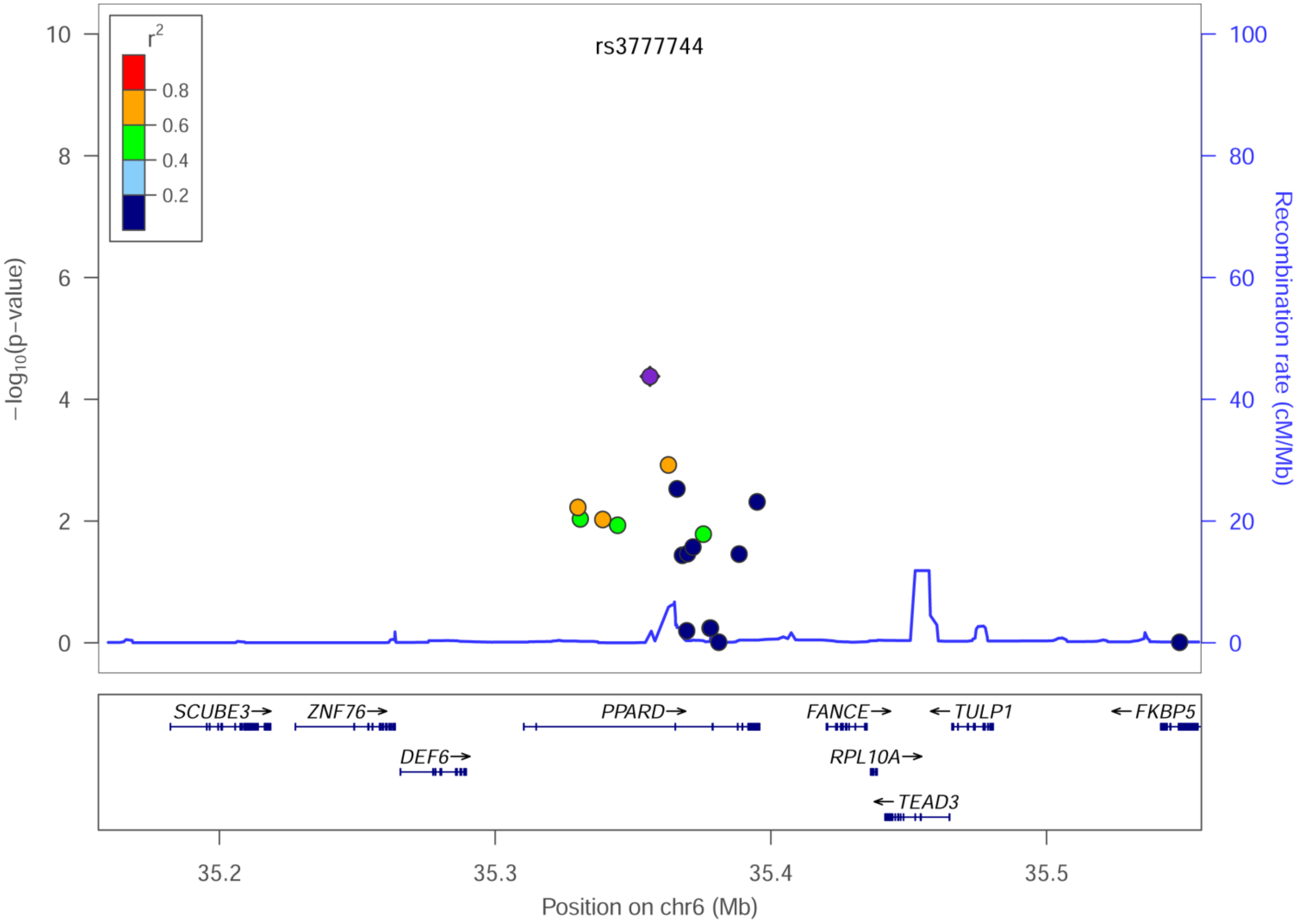
rs3777744
r2
0.8
0.6
0.4
0.2
−log10(p−value)
Recombination rate (cM/Mb)
0
2
4
6
8
10
0
20
40
60
80
100
SCUBE3→
ZNF76→
DEF6→
PPARD→
FANCE→
RPL10A→
←TEAD3
←TULP1
←FKBP5
35.2
35.3
35.4
35.5
Position on chr6 (Mb)

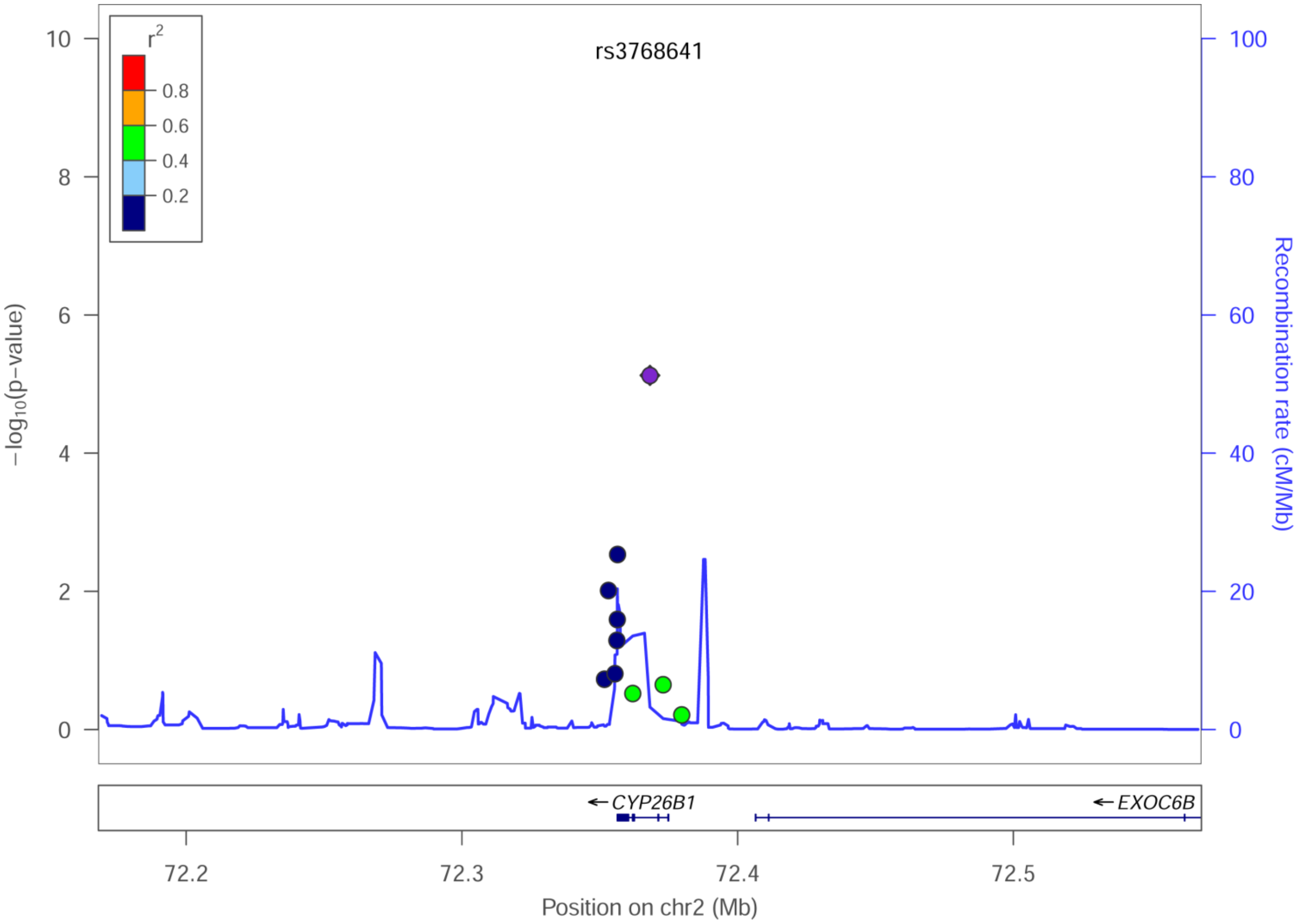

Figure S2: Regional plots of the five selected SNPs
